# Pro-arrhythmic effects of gain-of-function potassium channel mutations in the short QT syndrome

**DOI:** 10.1098/rstb.2022.0165

**Published:** 2023-06-19

**Authors:** J. C. Hancox, C. Y. Du, A. Butler, Y. Zhang, C. E. Dempsey, S. C. Harmer, H. Zhang

**Affiliations:** ^1^ School of Physiology, Pharmacology and Neuroscience, University of Bristol, Bristol BS8 1TD, UK; ^2^ School of Biochemistry, University of Bristol, Bristol BS8 1TD, UK; ^3^ Biological Physics Group, Department of Physics and Astronomy, University of Manchester, Manchester M13 9PL, UK

**Keywords:** arrhythmia, hERG, KCNQ1, KCNJ2, short QT syndrome, SQTS

## Abstract

The congenital short QT syndrome (SQTS) is a rare condition characterized by abbreviated rate-corrected QT (QTc) intervals on the electrocardiogram and by increased susceptibility to both atrial and ventricular arrhythmias and sudden death. Although mutations to multiple genes have been implicated in the SQTS, evidence of causality is particularly strong for the first three (SQT1−3) variants: these result from gain-of-function mutations in genes that encode K^+^ channel subunits responsible, respectively, for the I_Kr_, I_Ks_ and I_K1_ cardiac potassium currents. This article reviews evidence for the impact of SQT1-3 missense potassium channel gene mutations on the electrophysiological properties of I_Kr_, I_Ks_ and I_K1_ and of the links between these changes and arrhythmia susceptibility. Data from experimental and simulation studies and future directions for research in this field are considered.

This article is part of the theme issue ‘The heartbeat: its molecular basis and physiological mechanisms’.

## 1. Introduction

Ion channelopathies arise owing to acquired or genetic ion channel dysfunction. Channelopathies involving cardiac ion channels include long QT syndrome (LQTS), Brugada syndrome (BrS), catecholaminergic polymorphic ventricular tachycardia (CPVT), rare forms of familial atrial fibrillation (fAF), some forms of sick sinus syndrome (SSS) and the ‘short QT syndrome' (SQTS) [1,2]. A link between QT interval abbreviation (less than 400 ms) and increased risk of sudden cardiac death (SCD) in humans was made in 1993 [3], although an association between abbreviated ventricular repolarization and SCD was recognized earlier in some species of kangaroo [4,5]. Shorter than normal QT intervals occur in approximately 35% of male humans with idiopathic ventricular fibrillation (VF) [6]. Acquired QT interval shortening can occur with catecholamines, acetylcholine, hyperthermia, hypercalcaemia and anabolic steroid use [7–11]. As a distinct *congenital* condition, the SQTS is a relative newcomer, being first reported in 2000 [11]. The prevalence of congenital SQTS appears to be low [12,13]. However, the condition is strongly linked to both atrial and ventricular arrhythmias and, importantly, to sudden death [13–15], with approximately 40% of cases initially presenting with cardiac arrest [16]. SQTS is characterized by abbreviated rate-corrected QT (QT_c_) intervals and with poor rate-adaptation of the QT interval [13–15]. SQTS electrocardiograms (ECGs) often exhibit tall and narrow T waves, particularly in the precordial leads [13–15]. This article focuses on the congenital form of SQTS.

## 2. Diagnostic criteria and genotyping

As highlighted elsewhere [17], there are a number of factors that complicate the diagnosis of congenital SQTS. For instance, while approximately 0.1% of the population have QT_c_ intervals of less than 320 ms [18,19], it is difficult to state a clear cut-off between a healthy and pathologically short QT interval. Gollob and colleagues proposed diagnostic criteria that combine QT_c_ and J_point_−T_peak_ interval measurement with patient and family information [20]. Subsequently, the European Society of Cardiology (ESC) developed simplified criteria which suggest that a diagnosis of SQTS can be made with a QT_c_ interval of less than or equal to 340 ms [21]; a longer QT_c_ of less than or equal to 360 ms can be used if there is a family history of SQTS or SCD before 40 years of age, a pathogenic mutation has been identified, or there has been survival from ventricular tachycardia of fibrillation without structural heart disease [21]. A recent study of a Korean adult cohort with QT_c_ intervals of less than or equal to 340 ms found early repolarization, tall T waves, U waves and QT dispersion to be greater in those with a short QT interval compared to controls. A short QT interval, as defined in that study, was significantly associated with atrial fibrillation (AF) and ventricular arrhythmia/sudden arrest [22]. This appears to be consistent with the suggested ESC diagnostic QT_c_ values. It has been highlighted that the method of QT correction to derive QT_c_ values can influence diagnosis and it follows that measurements at low/resting heart rates are likely to be particularly useful [17,23]. Notably, it is not possible to rely strongly on the identification of pathogenic mutations in diagnosing SQTS, because genotyping is successful in less than 30% of cases [13,20]. A significant question arises as to whether or not diagnosis should be limited to a ‘pure' SQTS phenotype or whether it should take into account evidence that SQTS may sometimes be part of an ‘overlap syndrome', coexisting with BrS, SSS or early repolarization syndrome (ERS) phenotypes [12,24–26]. This issue is not merely of theoretical interest: it has practical implications. Nine different genes have been implicated in the SQTS (table 1 and [17]), but a recent study has suggested that only four of these have adequate evidence to be causally linked to SQTS [31]. In that study, independent teams studied the evidence for gene involvement using the ClinGen gene curation framework; as a result of their evaluations *KCNH2* (also known *as hERG; human Ether-à-go-go Related Gene*) was definitively linked to SQTS, while *KCNQ1*, *KCNJ2* and *SLC4A3* were considered to have moderate to strong evidence for causal involvement [31]. In highlighting a ‘disputed' classification for *CACNA1C, CACNA2D1, CACNB2* and *SCN5A*, these authors noted a lack of an SQTS phenotype in isolation for patients with mutations in these genes, as a BrS ECG pattern occurs accompanied by abbreviated QT intervals [31]. Mutations in *SLC22A5* (associated with primary systemic carnitine deficiency and QT abbreviation) were argued to cause a metabolic SQTS-mimic in which QT abbreviation occurs that is reversible on carnitine supplementation [31]. A recent investigation that aimed to identify ECG features for differentiation of SQTS in patients of less than 20 years of age has highlighted that QTc and J_point_-T_peak_ values (obtained with Bazett's correction) of less than 316 ms and 181 ms, respectively, and the presence of early repolarization may aid identification of SQTS in children and adolescents [32]. The concurrence of abbreviated QT_c_ interval with early repolarization noted in this report [32] highlights the potential significance of overlapping phenotypes. From the perspective of ion channel biology, it is unsurprising that mutations can lead to overlapping phenotypes, as the net consequences of an ion channel mutation will depend on the precise changes to current amplitude and kinetics that occur, and this could lead to a continuum of effects. Nevertheless, the remainder of this review will principally focus on links between *KCNH2*/*hERG*, *KCNQ1* and *KCNJ2* and short QT syndrome as these potassium channel genes can be causally linked to SQTS with a high degree of confidence. Of successfully genotyped SQTS variants involving mutations to K^+^ or Ca^2+^ channel genes, it has been estimated that more than 80% of patients had mutations to these genes (55.5% had *hERG* mutations, 11.1% had *KCNQ1* mutations and 14.8% had *KCNJ2* mutations) [33]. Thus, consideration of K^+^ channel mutations linked to SQTS covers the majority of successfully genotyped cases.

**Table 1.** A list of genes and associated mutations that have been linked to congenital SQTS. (The table is modified from [13] and [17], with additional mutations reported in [27–30]. ♂, inherited from father; ♀, inherited from mother; * truncation mutant. For all SQTS mutations to K^+^ channels discussed in this article, patients were heterozygous for the identified mutations.)

SQT subtype	*gene* and gene product	channel (subunit)	mutation (amino acid change)	gain/loss function
SQT1	*KCNH2* (hERG/Kv11.1)	*I*_Kr_ (*α* [pore-forming] subunit)	E50D	gain-of-function
			I560T	gain-of-function
			N588K	gain-of-function
			T618I	gain-of-function
			S631A	gain-of-function
			R1135H	gain-of-function
SQT2	*KCNQ1* (KCNQ1/Kv7.1/KvLQT1)	*I*_Ks_ (*α* subunit)	V141M	gain-of-function
			R259H	gain-of-function
			F279I	gain-of-function
			F279C	gain-of-function
			A287T	gain-of-function
			V307L	gain-of-function
SQT3	*KCNJ2* (Kir2.1)	*I*_K1_ (*α* subunit)	D172N	gain-of-function
			E299V	gain-of-function
			M301K	gain-of-function
			K346T	gain-of-function
SQT4	*CACNA1C* (Ca_V_1.2)	L-type *I*_Ca_ (*α* subunit)	A39V	loss-of-function
			G490R	loss-of-function
			K800T	loss-of-function
			R1973P	loss-of-function
			R1977Q	loss-of-function
SQT5	*CACNB2b* (*β*_2b_ subunit)	L-type *I*_Ca_ (accessory subunit)	S480L	loss-of-function
			S481L	loss-of-function
SQT6	*CACNA2D1* (*α*2*δ*1 subunit)	L-type *I*_Ca_ (accessory subunit)	S755T	loss-of-function
SQT7	*SCN5A* (Nav1.5)	*I*_Na_ (canonical *α* subunit)	R689H	putative loss-of-function
SQT8	*SLC4A3* (anion exchanger)	anion exchanger AE3	R370H	loss-of-function
SQT9	*SCN10A* (Nav1.8)	*I*_Na_ (non-canonical *α* subunit	G805V	presumed loss-of-function (functional data required)
**other**				
primary carnitine deficiency	*SLC22A5* (OCTN2)	OCTN2 carnitine transporter	W62X ♂ R471 ♀	loss-of-function
			R471 + null	
			R289*	

## 3. The SQT1 variant and mutations to *hERG*

**Figure 1.**
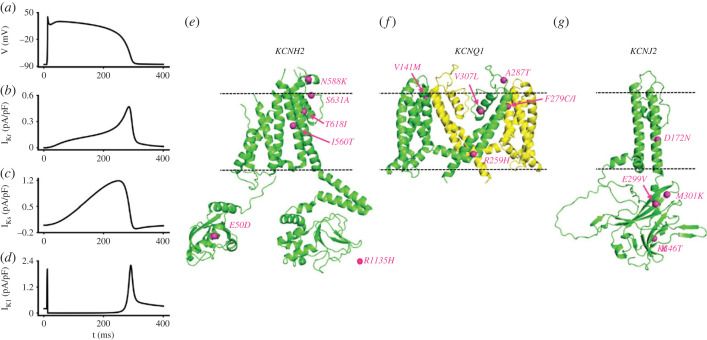
Potassium ion channels affected in SQTS variants 1–3. Panels (*a*–*d*) show simulated profiles of the three key repolarizing currents affected in SQT1, SQT2 and SQT3. (*a*) Shows a ventricular AP (elicited at stimulation rate of 1 Hz) from the ten Tusscher and Panfilov ventricular cell model [40]. Panel (*b*) shows corresponding time-course of I_Kr_, (*c*) shows corresponding time-course of I_Ks_, (*d*) shows corresponding time-course of I_K1_. (*e*–*g*) illustrate the locations of K^+^ channel SQTS mutants within the channel structures (identified by gene name). Mutants are highlighted on a single channel subunit (green). The *KCNH2* structure in (*e*) is from PDB:5VA2 ([41]; the C terminal cytoplasmic domain containing R1135 is not present in the structure). For *KCNQ1,* all SQTS mutants to date locate to the membrane domain and only this is shown in (*f*)—a second subunit (yellow) illustrates the manner in which subunits are assembled (as tetramers) in the membrane (all channels shown assemble as tetramers). The KCNQ1 structure is from PDB:6V00 [42]; and in (*g*) the *KCNJ2* derived Kir2.1 protein is an AlphaFold structure [43] with disordered cytoplasmic loops removed. (Online version in colour.)

*hERG* encodes a protein responsible for the pore-forming subunit of tetrameric I_Kr_ channels [34,35]. The potassium current carried by hERG and native I_Kr_ channels is characterized by very rapid, voltage-dependent inactivation that limits current at positive membrane potentials [34–37]. Consequently, hERG/I_Kr_ normally contributes little to early repolarization of ventricular action potentials (APs), with its contribution growing as the AP plateau descends and inactivation is relieved ([36,38,39] and figure 1*a,b*). The ability of hERG channels to generate rapid outward current transients in response to depolarizations applied late during AP repolarization/early in diastole means that I_Kr_ can also provide some protection against unwanted premature excitation [44].

The first genetically identified form of SQTS (the ‘SQT1' variant) was reported in 2004 [45]. Candidate gene screening identified heterozygous base substitutions in members of two different families that led to the same missense mutation (N588K) in the external S5-Pore linker region of the channel (figure 1*e*). Members of both families had markedly abbreviated QT intervals (QT_c_ of less than 300 ms) and atrial and ventricular arrhythmias; there were instances of sudden death without obvious structural abnormalities [45]. A subsequent study identified the same mutation in a different family with paroxysmal AF, abbreviated atrial and ventricular refractory periods and inducible VF [46]. The S5-Pore linker region of hERG is thought to be involved in the channel's rapid inactivation process [47–49] and patch clamp analysis of the N588K mutation has demonstrated that it results in a very marked rightward voltage shift in inactivation [50,51]. The net consequence of this is that hERG/I_Kr_ current rises much earlier during the ventricular AP, as it is not limited by the rapid inactivation that normally occurs. A larger outward current occurring earlier during the AP [45,50,51] results in AP abbreviation [52–55]. The N588K hERG mutation has been found in approximately 18.5% of genotyped SQTS cases [33]. Fourteen years after identification of the N588K mutation in SQT1, a pore mutant that produces a very similar attenuation of inactivation was reported [56]. The S631A mutation (figure 1*e*) had been studied previously in the investigation of the inactivation mechanism of hERG [57]. Its clinical relevance was established in a family in which a 6-year-old girl was screened following sudden death of a cousin [56]. She was found to have the S631A mutation and a QT_c_ interval of less than 320 ms. Her father and sister also had the mutation and abbreviated QT_c_ intervals (less than or equal to 340 ms). The girl was asymptomatic until a much later episode of syncope, as a result of which she received an implantable cardioverter defibrillator (ICD) [56]. Like the N588K mutation, S631A-hERG shows a profound attenuation of inactivation over physiologically relevant voltages [57] and under ventricular AP voltage clamp this produces an inverted **‘**U' or domed shape current [58,59].

The most commonly identified SQTS (and SQT1) mutation to date is the T618I hERG mutation, which has been found in multiple SQTS families and accounts for 25.9% of genotyped cases [33,60]. The T618 residue is located at a highly conserved site in the pore-loop of the hERG channel (figure 1*e*) and is strongly associated with ventricular arrhythmia, although in contrast with N588K it has not yet been linked to AF [33,61]. From patch clamp data obtained at room temperature, it has been suggested that negatively shifted voltage-dependent and accelerated time dependent activation contribute to the gain-of-function effect of the T618I hERG mutation [33]. However, data at physiological/near physiological temperature have shown a modest positive voltage-shift in activation [62–64] and an attenuation of voltage-dependent inactivation, albeit to a more modest extent than occurs for S631A or N588K mutations which is likely to substantially underlie the gain-of-function phenotype [60,63,64]. The current generated through T618I hERG during APs appears quite sensitive to the profile of the AP waveform and recent AP clamp data raise the possibility that the lack of reported AF with this mutation may reflect a reduction in gain-of-function during atrial compared to ventricular AP waveforms [64]. The mutation may also promote ventricular-Purkinje fibre differences in repolarizing I_Kr_, which may in turn contribute to U waves seen in carriers of this mutation [33,64]. Both T618I and N588K mutations also alter the generation of rapid transient currents in response to premature depolarizing stimuli [64,65].

The I560T hERG mutation was reported in a 64-year-old man who presented with palpitations and near syncope owing to AF and atrial flutter; his brother and father had died suddenly. Genetic screening revealed the I560T mutation in the S5 domain of hERG (figure 1*e*); the mutation was absent in genomic DNA of 200 controls [12]. This mutation leads to a positive shift in the voltage-dependence of inactivation and slowed time-course of inactivation and accelerated activation, also to increased ‘window' current as well as a modest decrease in K^+^ over Na^+^ selectivity [12,66]. Examination of the location of the I560 residue in the hERG protein structure suggested that the residue is oriented away from the pore towards membrane lipid, but it may interact with adjacent residue M561 and thereby influence interactions between S5 and the pore helix [66]. Two further SQTS-linked hERG mutations, E50D and R1135H have also been reported [20,67,68]. The N terminal E50D mutation (figure 1*e*) was observed in a 22-year-old man who lost consciousness when driving. He had a QT_c_ interval of 366 ms, with poor rate adaptation of the QT interval [67]. The E50D mutation has been suggested to increase hERG ‘tail' current magnitude, slow deactivation time-course and produce a modest (approx. 11–12 mV) positive shift in voltage-dependent inactivation [69]. The C terminal R1135H mutation (figure 1*e*) was found in a 34-year-old man with a mixed BrS/SQTS phenotype (QT_c_ interval of 329 ms). His mother and brother had QT_c_ intervals of less than 380 ms; his brother had a non-documented arrhythmia and his mother had bradycardia [70]. The R1135H mutation was described as increasing hERG current amplitude and slowing deactivation [70]. *In silico* simulations have demonstrated that fewer channels close during diastole with slowed deactivation with this mutant; this increases the contribution of hERG/I_Kr_ early during APs and may increase the likelihood of all-or-none right ventricular repolarization [24]. The R1135H example nicely illustrates the potential for BrS/SQTS overlap owing to a mutation's effects on current kinetics. It is notable in this regard that other hERG mutations that increase hERG current, but do not produce voltage-shifts in inactivation kinetics, have been reported in BrS patients with QT_c_ intervals less than 390 ms [71].

**Figure 2.**
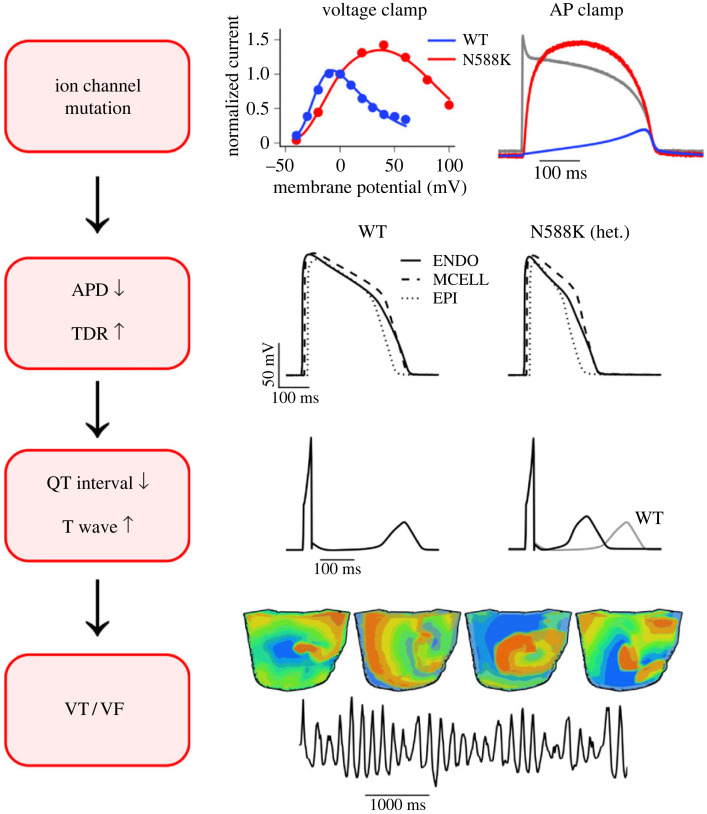
Summary of proarrhythmic effects of the N588K hERG mutation. Schematic diagram summarizing main proarrhythmic mechanisms of N588K hERG mutation in SQT1, identified from experiments on recombinant channels and computer simulations. Upper panels show shifted current voltage relation (left panel) and augmented current and altered current profile during ventricular AP (right panel). In simulations of human ventricular APs, the changes to I_Kr_ lead to abbreviated AP duration (APD) in epicardium (EPI), midmyocardium (MCELL) and endocardium (ENDO) and to augmented transmural dispersion of repolarization (TDR) [55,74]. At the tissue level, the QT interval becomes abbreviated and T wave amplitude increased. These changes increased susceptibility of ventricular tissue to ventricular tachycardia (VT)/fibrillation (VF) (bottom panels). Figure is reproduced from [17], under a CC BY Creative Commons 4.0 licence (https://creativecommons.org/licenses/by/4.0/legalcode). (Online version in colour.)

Computational simulations of the effects of the N588K mutation have provided insight into arrhythmia mechanisms in SQT1 [54,55,72–74]. As S631A-hERG exhibits comparable attenuation of inactivation to that seen with N588K [75], results of simulations based on N588K-hERG data are likely to also apply in the setting of the S631A mutation. In ventricular simulations, incorporation of N588K-attenuated inactivation led to marked abbreviation of AP duration (APD) and effective refractory period (ERP) [55]. In multicellular simulations, the N588K mutation augmented *δ*V (membrane voltage heterogeneity) in localized regions of the ventricular wall, contributing to T wave changes and facilitating vulnerability to re-entry. Simulations at two-dimensional and three-dimensional levels highlighted a marked decrease in the minimal substrate size required to sustain re-entry, resulting in increased lifespan durations for induced spiral or scroll waves ([55] and figure 2). Incorporation of N588K into atrial cell and tissue simulations has also shown mutation-induced abbreviation of APD and ERP, shortened excitation wavelength and increased scroll wave lifespan and dominant frequency [76,77]. In electromechanically coupled human ventricle cell models, incorporation of the N588K mutation reduced [Ca^2+^]_i_ transients and contractile force, while in three-dimensional ventricle models the timing of maximum deformation (contraction) was delayed by N588K compared to wild-type I_Kr_ [72]. A subsequent clinical report was published in which SQTS patients were investigated with Doppler imaging and speckle tracking electrocardiography; this showed some decrease in left ventricular contraction and increased mechanical dispersion in SQTS patients compared to healthy controls [78–80]. Computational analysis of the effects of the I560T mutation has also shown abbreviation of ventricular APD and simulated QT intervals [12]. In tissue simulations, sustained re-entry was not observed under control conditions, but incorporation of I560T-induced effects led to sustained spiral wave re-entry [12].

## 4. The SQT2 variant and mutations to *KCNQ1*

**Figure 3.**
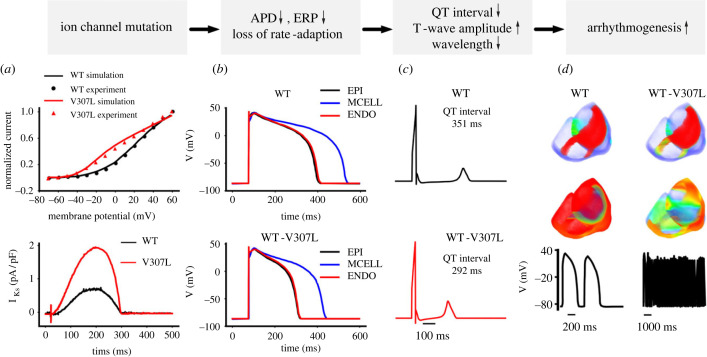
Summary of proarrhythmic effects of the V307L KCNQ1 mutation. Panel (*a*) shows changes to I_Ks_ under conventional voltage clamp (upper panel) and AP voltage clamp (lower panel), showing gain-of-function consequences of the V307L KCNQ1 mutation. Panel (*b*) shows simulated changes to ventricular AP durations in epicardium (EPI), midmyocardium (MCELL) and endocardium (ENDO); upper panel shows control APs and lower panels show abbreviated APs when effects of V307L mutation on I_Ks_ are incorporated. Panel (*c*) shows simulated pseudo-ECG, demonstrating QT interval abbreviation owing to V307L-KCNQ1. Panel (*d*) shows consequences at whole ventricle levels of the V307L mutation (spiral wave re-entry at two-dimensional level, scroll wave re-entry at three-dimensional level), with the tissue able to sustain high-frequency excitation. The figure is based on and modified from [92], under a CC BY Creative Commons 4.0 licence (https://creativecommons.org/licenses/by/4.0/legalcode)/. (Online version in colour.)

The KCNQ1 protein (also known as KvLQT1 and Kv7.1) combines with KCNE1 (alternative nomenclature minK) to form functional I_Ks_ channels [81,82]. In undiseased human cardiomyocytes, I_Ks_ exhibits slow activation and fast deactivation kinetics [83] and is recognized to be a key component of **‘**repolarization reserve' ([84–86] see also figure 1*a*,*c*). Its contribution to AP repolarization is augmented under conditions of *β* adrenoceptor activation [87]. Loss-of-function KCNQ1 mutations are well-established to underlie the LQT1 form of LQTS [88]. The first KCNQ1 mutation identified in the SQTS (SQT2) was V307L, which lies within the P-loop of the KCNQ1 protein (figure 1*f*); the mutation was found in a 70-year-old male who experienced an episode of aborted sudden death [89]. When co-expressed with KCNE1, V307L-KCNQ1 channels have been shown to pass current with negative-shifted voltage-dependent activation, modestly accelerated activation time-course and slowed deactivation time-course [89,90]. The augmented I_Ks_ that results from these changes has been shown to be causally linked to accelerated ventricular repolarization and arrhythmogenesis *in silico* ([91,92] and figure 3). The V141M mutation produces a more severe alteration to I_Ks_ and was first reported in a case of *in utero* bradycardia and irregular rhythm [93]. The V141 residue is located in the S1 domain of the KCNQ1 protein (figure 1*f*). The V141M mutation produces an instantaneous component of I_Ks_ and computational modelling showed ventricular AP abbreviation and cessation of pacemaking activity [93]. Subsequent to the original report, the V141M mutation has been confirmed to be linked to a mixed AF/sinus bradycardia phenotype [12,93–95]. In an *in silico* comparison of the consequences of the V307L and V141M mutations, both led to atrial AP abbreviation with a greater effect of V141M than V307L. However, only the V141M KCNQ1 mutation significantly altered sinoatrial nodal pacemaking rate, because it has a more marked effect on I_Ks_ over the diastolic depolarization membrane potential range [96]. Moreover, although both V307L and V141M mutations promoted re-entry in atrial tissue simulations, they produced different effects on the steepness of restitution of atrial AP duration, with V141M leading to stable, stationary spiral waves and V307L leading to non-stationary unstable waves [96].

The F279I mutation occurs at a residue located in the S5 segment of the KCNQ1 protein (figure 1*f*) and was identified in a 23-year-old male with a QT_c_ interval of 356 ms and a familial history of sudden death [97]. Functional analysis of F279I KCNQ1 in the presence of KCNE1 revealed a negative shift in the voltage dependence of I_Ks_ activation and accelerated activation kinetics. Further insight came from co-immunoprecipitation and FRET measurements, which showed that the ability of KCNQ1 to coassemble with KCNE1 is impaired by the F279I mutation [97]. Computational modelling demonstrated AP abbreviation as a result of F279I-induced changes to I_Ks_ [97]. More recently, a second mutation (F279C) at the same residue position (figure 1*f*) was identified from genetic screening of a 10-year-old girl who experienced, but survived sudden cardiac arrest during a vacation [27]. Her initial in-hospital ECG showed a QT_c_ of 383 ms, but telemetry and serial ECG measurement showed a minimal QT_c_ of 344 ms. Genetic screening identified three variants of uncertain significance to *RYR2, TTN* and *KCNQ1*. The *RYR2* variant was predicted to be tolerated by *in silico* prediction tools and was found also to be present in some population databases. The *TTN* variant was also identified in a population database [27]. The KCNQ1 variant was not found in any population database and had not been reported previously in disease. F279 has been shown to interact with F232 in a study employing cysteine mutagenesis that showed F232 and F279 are jointly responsible for gating modulation of KCNQ1 by KCNE1 [98]. In the presence of KCNE1, F279C channels exhibited negatively shifted voltage-dependent activation compared to wild-type KCNQ1 [98]. This is consistent with a gain-of-function effect [27].

The R259H KCNQ1 mutation (figure 1*f*) was first reported in a study of suspected SQTS in which genetic screening was conducted of 42 probands and three affected relatives of deceased probands. The affected individual had experienced an aborted cardiac arrest and had a QT_c_ interval of 316 ms [16]. It was also subsequently reported in a Chinese SQTS proband from screening 25 probands and family members for mutations to genes for ion channels that influence ventricular repolarization [99]. In patch clamp experiments, R259H KCNQ1 augmented I_Ks_ density and accelerated activation time-course and slowed deactivation, but did not significantly alter the voltage dependence of current activation [99]. The A287T KCNQ1 mutation was identified from genetic testing of a female patient who had survived cardiac arrest following VF [28]. Three years after implantation of an ICD an episode of VF occurred that was successfully cardioverted [28]. Electrophysiological measurements from A287T-KCNQ1 and KCNE1 coexpressed in *Xenopus* oocytes showed increased current amplitude and accelerated activation time-course compared to wild-type KCNQ1 [28]. Structural analysis showed that the position of the A287 residue (at the top of S5; figure 1*f*) allows it to form a hydrogen bond with a hydroxyl group of residue T322 at the top of S6, which may influence gating [28]. The results of molecular dynamics simulations suggested that the A287 residue is important for both correct selectivity filter structure and interaction with KCNE1 [28]. A further KCNQ1 mutation, I274V, *may* also be associated with SQTS [100]. This mutation was identified from genetic screening of genomic DNA from 201 cases of sudden infant death syndrome (SIDS) [101]. Functional analysis showed increased magnitude of I274V KCNQ1 + KCNE1 current compared to that from channels incorporating wild-type KCNQ1, while it also produced a modest acceleration of current activation and slowed current deactivation [100]. Incorporation of the effects of this mutation in ventricular AP simulations led to marked AP abbreviation [100]. These observations are strongly consistent with an SQTS phenotype; however, the absence of ECG data preclude definitive diagnosis as SQTS.

## 5. The SQT3 variant and mutations to *KCNJ2*

The inwardly rectifying K^+^ current, I_K1_, plays important roles in maintaining the resting potential and in terminal repolarization of the ventricular AP [81,82]: as the amplitude of I_Kr_ declines, that of I_K1_ increases dominating the final repolarization phase ([86,102] figure 1*a*,*d*). The channels that mediate I_K1_ are comprised of Kir2.x subunits, with *KCNJ2*-encoded Kir2.1 strongly expressed in both atria and ventricles [103,104]. Loss-of-function *KCNJ2* mutations underlie Andersen-Tawil syndrome (also known as the LQT7 form of LQTS [105]). The first *KCNJ2* mutation reported to underlie the SQT3 form of SQTS was identified in a 5-year-old girl from whom an abnormal ECG was obtained during routine physical examination [53]: a short QT_c_ interval of 315 ms and a narrow, asymmetric T wave with a rapid terminal phase. The girl's father had a history of palpitations on presyncopal events. Genetic analysis revealed no mutations in either *hERG* or *KCNQ1*, but a *KCNJ2* variant was identified that led to a missense mutation (D172N) in Kir2.1 [53]. The inward rectification of the potassium current carried by Kir2.1 arises owing to a voltage-dependent block of the channel by polyamines and Mg^2+^ ions and the D172 residue (figure 1*g*) is implicated in Mg^2+^ and polyamine binding [106]. The D172N mutation impairs this process and results in increased outward current, with a modest positive shift in the voltage at which peak outward current occurs, though does not eliminate current rectification entirely [53,106,107]. Ventricular AP simulations in the study reporting the D172N variant showed steeper APD restitution and AP abbreviation involving increased steepness of terminal repolarization [53]. One-dimensional tissue strand simulations reproduced the T wave morphology seen in the proband [53]. Subsequent independent simulations at one- to three-dimensional tissue levels showed that incorporation of D172N mutant I_K1_ abbreviated APD and ERP and steepened both APD and ERP restitution. Tissue excitability was increased at high rates [108]. Temporal vulnerability to initiation of re-entry was enhanced, while minimal substrate size required for re-entry maintenance was decreased for the D172N mutant [108]. Further simulations using electro-mechanically coupled ventricle models showed some theoretical potential for the D172N mutation to adversely influence contractile force [72].

The M301K mutation in Kir2.1 (figure 1*g*) was found in an 8-year-old girl with a QT_c_ interval of 194 ms and AF that was unresponsive to intravenous procainamide [109], who underwent electrical cardioversion. AF and VF were inducible on electrophysiological testing [109]. The M301 residue is located in the C terminus of the Kir2.1 protein, is highly conserved and the M301K mutation was absent in 400 control alleles [109]. When the M301K protein was expressed alone, it failed to produce functional channels, but when co-expressed with wild-type Kir2.1 (which reflected the heterozygous status of the patient) outward current was increased over a wide range of potentials [109], with a more marked loss of rectification than reported earlier for the D172N mutation [53,107]. Recently, the predominant microRNA (miR) in the heart, miR1, has been found to bind to Kir2.1 and suppress I_K1_ carried by wild-type Kir2.1 at sub picomolar concentrations; this effect was absent for channels incorporating M301K Kir2.1 [110]. This constitutes a potential additional factor that contributes to the gain-of-function effect of this SQTS mutation.

The K346T mutation (figure 1*g*) was found in 9-year-old identical twins with epilepsy and behavioural impairment who also showed an abbreviated QT_c_ interval in ECG measurements (QT_c_ of 331 ms), with narrow peaked T waves [111]. The K346 residue is highly conserved and the mutation was absent in 400 ethnically matched control chromosomes and in large polymorphism databases [111]. Recordings of Kir2.1 currents from *Xenopus* oocytes were made using high (90 mM) external potassium and the K346T mutation was found to augment inward current under these conditions. Additional measurements made using mammalian cell line expression and more physiological recording conditions showed increases to both inward and outward currents for K346T Kir2.1 channels. The increased current occurred without significant alterations to unitary slope conductance [111]. This mutation also appears to increase Kir2.1 protein stability by decreasing Kir2.1 ubiquitination and degradation [111].

The E299V Kir2.1 mutation (figure 1*g*) was found in an 11-year-old boy with recurrent paroxysmal AF and mild left ventricular dysfunction [112]. Holter monitoring showed an average ventricular response of 98 beats per minute during paroxysms of AF. At a heart rate of 60 beats per minute, the QT interval was 200 ms and the QT-heart rate relation was flat, showing poor rate adaptation of the QT interval [112]. The E299V mutation was absent in the boy's parents and in 400 controls [112]. Expression and subcellular distribution of the E299V protein were similar *in vitro,* and there was no sign of any trafficking defect for E299V Kir2.1. However, in patch clamp analysis, E299V channels (expressed either alone or with wild-type Kir2.1) showed large increases in outward current over most of the physiological range of membrane potentials. AP voltage clamp experiments showed large Kir2.1 current increases at both APD_5_ and APD_50_ timepoints [112]. Ventricular AP simulations comparing effects of the E299V and D172N mutations showed much greater APD abbreviation with the former mutation and a profound flattening of the APD_90_ rate relationship, consistent with the marked effects of E299V throughout the AP [112]. In ventricular tissue simulations of heterozygous E299V Kir2.1 the S1-S2 protocol interval that produced re-entry was shifted to towards shorter intervals and sustained re-entry with twice the frequency possible for wild-type tissue was observed. Introduction of the mutation into three-dimensional ventricle simulations introduced left-right asynchronicity of excitation (excitation of the two ventricles via conduction of the sinus beat via the His bundle and distal Purkinje fibre system was synchronous in the wild-type condition, but for the right ventricle was delayed in the E299V condition). In atrial simulations profound AP shortening and flat APD­_90_-rate relations were observed [112]. It proved difficult to elicit re-entry in tissue simulations using an S1-S2 protocol with wild-type Kir2.1, but with E299V present windows of vulnerability to re-entry were seen. For both the E229V and D172N Kir2.1 mutations the causal link between mutation-induced changes to I_K1_ and abbreviated repolarization has also been verified experimentally using the dynamic clamp technique, in which application to cardiomyocytes in real-time of mutation-induced changes to I_K1_ has been shown to abbreviate APD [113,114].

## 6. Evidence for an overlap syndrome involving increased K_ATP_ channel activity?

A recent study highlights the potential for SQTS to overlap with ERS and raises the possibility that other cardiac K^+^ currents than I_Kr_, I_Ks_ and I_K1_ might contribute to SQTS [115]. An adult (27 years) male presented at the doctor owing to frequent episodes of syncope several weeks following electrocution at work. ECG analysis showed an ERS phenotype together with an abbreviated QT_c_ interval of 326 ms, consistent with concomitant SQTS. Programmed electrical stimulation evoked arrhythmias and arrhythmia inducibility was exacerbated by procainamide administration, necessitating electrical cardioversion [115]. On genetic analysis (next generation sequencing), six heterozygous exonic mutations were found, three of which were predicted to be damaging: R3634D in *ANK2* (Ankyrin-B); D26N in *PKP2* (Plakophilin 2) and R663C in *ABCC9* (SUR2). A *KCNA5* encoded A251T mutation to Kv1.5 was predicted to be tolerated and a short deletion mutation found in *HCN2* would not be anticipated to influence repolarization of ventricular tissue. Patient derived induced pluripotent stem cells (iPSCs) were produced from fibroblasts and differentiated into cardiomyocytes (iPSC-CMs). Measurements from these were compared with those from a wild-type iPSC-CM line. Field potential recordings showed an absence of a pseudo-QRS complex in myocytes derived from the patient and voltage clamp analysis showed a significant reduction in fast Na^+^ current (I­_Na_). This may have been linked to the *PKP2* mutation in the patient [115]. Importantly, the duration of spontaneous APs from patient derived myocytes was markedly shorter than that from control myocytes [115]. This occurred despite a reduced spontaneous rate (and hence longer cycle length); at a consistent pacing rate, the extent of AP abbreviation might be greater than that reported from spontaneous AP recording. SUR2 combines with Kir6.x channels to form K_ATP_ channels [81,82], which are usually silent under normoxic conditions. The abbreviated APs in iPSC-CMs from the patient could be prolonged by the application of the K_ATP_ inhibitor glibenclamide. This suggests that the SUR2 R663C mutation could have led to constitutively active K_ATP_ channels in the patient, which led to QT interval abbreviation [115]. Direct recordings of K_ATP_ current in expression studies of channels incorporating the SUR2 R663C mutation would be valuable to investigate further the phenotype observed in this study. The reason why syncope only appeared after electrocution, despite the presence of these mutations throughout life, remains unexplained. Nevertheless, this recent report provides interesting evidence implicating K_ATP_ channels in producing an abbreviated QT_c_ interval phenotype and this warrants further study.

## 7. Insights into arrhythmia mechanisms from experimental models

The foregoing discussion has highlighted pro-arrhythmic mechanisms in K^+^ channel mutation linked SQTS identified through computational modelling. It is valuable also to consider insights into arrhythmogenesis in SQTS from experimental studies. Years before the possibility emerged that mutations to K_ATP_ channel subunits might contribute clinically to an SQTS phenotype, the K_ATP_ channel opener pinacidil was used as a tool to produce an experimental SQTS phenotype in the canine ventricular wedge preparation and rabbit perfused hearts [116–118]. Pinacidil shortened repolarization, abbreviated ERP, increased transmural heterogeneity of repolarization and increased susceptibility to ventricular arrhythmia [116–118]. The hERG/I_Kr_ channel opener PD118057 produced similar effects in the canine ventricular wedge preparation [119]. The first genetic model of SQTS was the *reggae* zebrafish mutant, in which a mutation to the S4 domain of the zebrafish hERG homologue zERG (L499P) produced increased repolarizing current and abbreviated compound action potentials and QT_c_ intervals [120]. Analysis of alterations to current kinetics of the equivalent mutation (L532P) in hERG has shown a moderate positive shift in the voltage dependence of inactivation and alterations to activation/deactivation kinetics leading to a marked increase in repolarizing current and altered current timing during the ventricular AP [120,121]. To date, this mutation has not been detected in human SQTS.

A significant advance in recent years has been the development of a transgenic rabbit model of SQT1 produced through expression of a human transgene for N588K-hERG [122]. This model exhibits abbreviation of QT_c_ intervals, atrial and ventricular APs and of ERP. Ventricular arrhythmias could readily be induced in N588K-hERG expressing perfused hearts [122]. Intriguingly, some electrical remodelling of other currents was observed, including a decrease in I_K1_ and increase in I_Ks_ [122]. An increase in T wave height (as occurs in SQT1 patients) was not reported and while diastolic relaxation was enhanced altered systolic function was not observed [122]. Nevertheless, the SQT1 rabbit very largely recapitulates human SQT1 and highlights similar arrhythmia mechanisms to those predicted from simulations.

Human iPSC-CMs have been generated for both the N588K and T618I hERG SQT1 mutations [123–125]. A recognized limitation with iPSC-CMs is their relatively immature, spontaneously active phenotype, in which I_K1_ is small or absent [126,127]. Thus, while N588K-SQT1 hiPSC-CMs exhibit increased I_Kr_ and abbreviated APs, they are spontaneously active; abnormal Ca^2+^ transients after-depolarization events were seen in these cells that were worsened by carbachol application [123]. On the one hand, this highlights a potential trigger for arrhythmia under conditions of parasympathetic stimulation, while on the other, the relevance of such findings to what happens in mature cardiomyocytes is uncertain. Intriguingly, some remodelling of ion channel messenger RNAs (mRNAs) was found in N588K-hiPSC-CMs, with upregulation for *CACNA1C*, *hERG* and *KCND3 + KChIP2*, but no changes in mRNA for *KCNQ1* and *KCNJ2* [123]. Further work has been conducted using a combination of single cell recording and measurements from two-dimensional sheets of N588K-SQT1 hiPSC-CMs [125]. Patch clamp recording from single cells showed increased I_Kr_ and abbreviated repolarization and refractoriness. Consistent with prior simulation work and transgenic rabbit data, optical mapping experiments on SQT1 two-dimensional myocyte sheets highlighted abbreviated excitation wavelength, altered rate adaptation of APD and increased inducibility of spiral waves of increased stability and frequency [125]. It is reassuring that different experimental and simulation approaches have revealed similar mechanisms of proarrhythmia in this form of SQT1 [128]. Patient derived iPSC-CMs harbouring the T618I hERG mutation exhibit increased I_Kr_, abbreviated APs and increased beat-to-beat variability in spontaneous AP firing pattern (with regular spontaneous activity in control iPSC-CMs and AP bursts with interspersed pauses in T618I-hERG-hiPSC-CMs) [124]. Such spontaneous firing patterns have no direct correlate in mature cardiac myocytes. T618I-hiPSC-CMs exhibit significant remodelling in ion channel genes and it is notable that membrane expression of hERG protein was increased for T618I-hiPSC-CMs compared to controls [124].

## 8. Treatment

Given the risk of sudden death in SQTS, ICDs have been used extensively in management of the syndrome [14,129–132]. Potential T wave oversensing can be managed through device reprogramming [130]. However, although ICDs may prevent death they do not normalize repolarization or arrhythmia substrates. The mainstay of pharmacological treatment of SQTS is the Class Ia antiarrhythmic quinidine [13,132,133]. ESC guidelines from 2015 give a class IIb recommendation (may be considered) for quinidine and sotalol for patients with SQTS who qualify for an ICD but have a contraindication for or refuse ICD use [21]. These drugs may also be considered for use in asymptomatic patients with a family history of sudden death [21]. A recent (2022) update to these guidelines retains these recommendations for quinidine, though not for sotalol [134]. From an ion channel pharmacology perspective, both quinidine and sotalol should be effective at delaying repolarization through hERG/I_Kr_ block in forms of SQTS other than SQT1. However, sotalol and ibutilide were found to be ineffective in cases of SQT1, while quinidine normalized repolarization and protected against arrhythmia [45,135,136]. The ineffectiveness of pure class III hERG blocking drugs in SQT1 with severe hERG inactivation impairment can be explained by the dependence of these drugs on an intact inactivation process to bind optimally to the hERG channel [75,136–138]. By contrast quinidine and another class Ia drug disopyramide have comparatively little dependence on intact hERG inactivation to bind and are effective inhibitors of hERG channels with SQT1 linked mutations [60,63,66,75,137,139]. Quinidine has been found to be effective in experimental models of SQTS (reviewed in [13,128]) and in simulations is effective in prolonging repolarization across SQT1-SQT3 settings [140]. Multiple simulation and experimental studies have now been conducted that have investigated potential alternative/additional pharmacotherapeutic strategies in SQTS and detailed consideration of these is beyond the scope of this article. For further consideration of potential SQTS treatments, the reader is referred to [12–14,33,61,132,133,141].

## 9. Conclusion and future directions

The existence of SQT1–3 underscores the importance of I_Kr_, I_Ks_ and I_K1_ for human ventricular repolarization. Although the individual mutations considered in this review have distinct effects on the function of different K^+^ channels, common consequences of the different K^+^ channel gain-of-function mutations considered here are abbreviated ventricular repolarization and refractoriness and increased vulnerability to initiation and maintenance of re-entry. In cases where a channelopathy arises owing to mutations in identified ion channel genes, characterization of gene variants using a combination of heterologous expression and computational modelling is likely to continue to provide significant insight into whether or not an identified ion channel gene variant is pathogenic [142]. However, the relatively low success of targeted genetic screening in the SQTS indicates involvement of additional genes to those that are routinely screened for electrical disorders. It is possible that some SQTS cases may arise owing to mutations in genes that encode modifiers of channels involved in repolarization rather than ion channels themselves. Some cases may be polygenic. Such causes may not be readily identified through targeted gene screening and addressing this challenge may require the use of whole exome/genome approaches, followed by functional validation of putative modifier genes. The field is currently somewhat limited by a relative lack of genetically accurate animal models of different forms of SQTS. Ideally, future progress in this direction will involve targeted modification of endogenous channel genes. The use of such animal models will be important both to validate predictions from computational modelling, to investigate facets such as remodelling and effects of modifier genes that are difficult to study in heterologous expression and simulations alone, and to investigate novel therapeutic approaches. Patient-specific iPSC-CMs are likely to assume increasing importance in the interrogation of SQTS (and other primary electrical disorders), both in exploring causation and potential genetic correction. While the immaturity of iPSC-CMs and consequent spontaneous electrical activity at present impose some limitations on the interpretation of findings using patient-derived iPSC-CMs, at the single cell level at least, the lack of a stable resting potential can be remedied through electronic I_K1_ application with dynamic clamp [64,113,143]. The development of improved maturation approaches will ultimately be of high value in the employment of iPSC-CMs, not least in the interrogation of patient-specific treatment approaches and in the further exploration of novel approaches to modulating repolarization, such as miRNA treatment and optogenetics [144–146]. A recent systematic literature review has suggested the existence of some sex differences in SQTS features [147], specifically a predominance of syncope among males and a higher risk of arrhythmic events or SCD at diagnosis and during follow up in females. The role of sex hormones in modifying arrhythmia mechanisms/substrates in the SQTS remains to be investigated. Future systematic investigation of how identified SQT1-3 K^+^ channel mutations interact with autonomic modulation is also likely to be profitable.

## Data Availability

This article has no additional data.

## References

[1] Kim JB. 2014 Channelopathies. Korean J. Pediatr. **57**, 1-18. (10.3345/kjp.2014.57.1.1) 24578711 PMC3935107

[2] Campuzano O, Brugada R. 2009 Genetics of familial atrial fibrillation. Europace **11**, 1267-1271. (10.1093/europace/eup199) 19666641

[3] Algra A, Tijssen JG, Roelandt JR, Pool J, Lubsen J. 1993 QT interval variables from 24 hour electrocardiography and the two year risk of sudden death. Br. Heart J. **70**, 43-48. (10.1136/hrt.70.1.43) 8037997 PMC1025227

[4] Campbell TJ. 1989 Characteristics of cardiac action potentials in marsupials. J. Comp. Physiol. B **158**, 759-762. (10.1007/BF00693014) 2715459

[5] Rezakhani A, Webster JD, Atwell RB. 1986 The electrocardiogram of the eastern grey kangaroo (*Macropus giganteus*). Aust. Vet. J. **63**, 310-312. (10.1111/j.1751-0813.1986.tb08078.x) 3800805

[6] Viskin S, Zeltser D, Ish-Shalom M, Katz A, Glikson M, Justo D, Tekes-Manova D, Belhassen B. 2004 Is idiopathic ventricular fibrillation a short QT syndrome? Comparison of QT intervals of patients with idiopathic ventricular fibrillation and healthy controls? Heart Rhythm **1**, 587-591. (10.1016/j.hrthm.2004.07.010) 15851224

[7] Garberoglio L, Giustetto C, Wolpert C, Gaita F. 2007 Is acquired short QT due to digitalis intoxication responsible for malignant ventricular arrhythmias? J. Electrocardiol. **40**, 43-46. (10.1016/j.jelectrocard.2006.07.003) 16950330

[8] Bidoggia H et al. 2000 Sex differences on the electrocardiographic pattern of cardiac repolarization: possible role of testosterone. Am. Heart J. **140**, 678-683. (10.1067/mhj.2000.109918) 11011345

[9] Charbit B, Christin-Maitre S, Demolis JL, Soustre E, Young J, Funck-Brentano C. 2009 Effects of testosterone on ventricular repolarization in hypogonadic men. Am. J. Cardiol. **103**, 887-890. (10.1016/j.amjcard.2008.11.041) 19268751

[10] Bigi MA, Aslani A, Aslani A. 2009 Short QT interval: a novel predictor of androgen abuse in strength trained athletes. Ann. Noninvasive Electrocardiol. **14**, 35-39. (10.1111/j.1542-474X.2008.00271.x) 19149791 PMC6932133

[11] Gussak I, Brugada P, Brugada J, Wright RS, Kopecky SL, Chaitman BR, Bjerregaard P. 2000 Idiopathic short QT interval: a new clinical syndrome? Cardiology **94**, 99-102. (10.1159/000047299) 11173780

[12] Harrell DT et al. 2015 Genotype-dependent differences in age of manifestation and arrhythmia complications in short QT syndrome. Int. J. Cardiol. **190**, 393-402. (10.1016/j.ijcard.2015.04.090) 25974115

[13] Hancox JC, Whittaker DG, Du C, Stuart AG, Zhang H. 2018 Emerging therapeutic targets in the short QT syndrome. Expert Opin. Ther. Targets **22**, 439-451. (10.1080/14728222.2018.1470621) 29697308

[14] Maury P et al. 2008 Short QT syndrome. Update on a recent entity. Arch. Cardiovasc. Dis. **101**, 779-786. (10.1016/j.acvd.2008.08.009) 19059573

[15] Giustetto C et al. 2006 Short QT syndrome: clinical findings and diagnostic-therapeutic implications. Eur. Heart J. **27**, 2440-2447. (10.1093/eurheartj/ehl185) 16926178

[16] Mazzanti A et al. 2014 Novel insight into the natural history of short QT syndrome. J. Am. Coll. Cardiol. **63**, 1300-1308. (10.1016/j.jacc.2013.09.078) 24291113 PMC3988978

[17] Hancox JC, Whittaker DG, Zhang H, Stuart AG. 2019 Learning from studying very rare cardiac conditions: the example of short QT syndrome. J. Congenit. Cardiol. **3**, 1-15. (10.1186/s40949-019-0024-7)

[18] Anttonen O, Junttila MJ, Rissanen H, Reunanen A, Viitasalo M, Huikuri HV. 2007 Prevalence and prognostic significance of short QT interval in a middle-aged Finnish population. Circulation **116**, 714-720. (10.1161/CIRCULATIONAHA.106.676551) 17679619

[19] Dhutia H et al. 2016 The prevalence and significance of a short QT interval in 18,825 low-risk individuals including athletes. Br. J. Sports Med. **50**, 124-129. (10.1136/bjsports-2015-094827) 26400956

[20] Gollob MH, Redpath CJ, Roberts JD. 2011 The short QT syndrome: proposed diagnostic criteria. J. Am. Coll. Cardiol. **57**, 802-812. (10.1016/j.jacc.2010.09.048) 21310316

[21] Priori SG et al. 2015 ESC Guidelines for the management of patients with ventricular arrhythmias and the prevention of sudden cardiac death: The Task Force for the Management of Patients with Ventricular Arrhythmias and the Prevention of Sudden Cardiac Death of the European Society of Cardiology (ESC). Endorsed by: Association for European Paediatric and Congenital Cardiology (AEPC). Eur. Heart J. **36**, 2793-2867. (10.1093/eurheartj/ehv316) 26320108

[22] Kim D-Y et al. 2021 Long-term prognosis of short QT interval in Korean patients: a multicenter retrospective cohort study. BMC Cardiovasc. Disord. **21**, 17. (10.1186/s12872-020-01824-3) 33407155 PMC7788900

[23] Providência R, Karim N, Srinivasan N, Honarbakhsh S, Ferreira MJV, Gonçalves L, Marijon E, Lambiase PD. 2017 Impact of QTc formulae in the prevalence of short corrected QT interval and impact on probability and diagnosis of short QT syndrome. Heart **104**, 502-508. (10.1136/heartjnl-2017-311673) 28954836

[24] Wilders R, Verkerk AO. 2010 Role of the R1135H KCNH2 mutation in Brugada syndrome. Int. J. Cardiol. **144**, 149-151. (10.1016/j.ijcard.2008.12.177) 19174314

[25] Antzelevitch C et al. 2007 Loss-of-function mutations in the cardiac calcium channel underlie a new clinical entity characterized by ST-segment elevation, short QT intervals, and sudden cardiac death. Circulation **115**, 442-449. (10.1161/CIRCULATIONAHA.106.668392) 17224476 PMC1952683

[26] Watanabe H et al. 2010 High prevalence of early repolarization in short QT syndrome. Heart Rhythm **7**, 647-652. (10.1016/j.hrthm.2010.01.012) 20206319

[27] Schneider K, Parrott A, Spar D, Knilans T, Czosek R, Miller E, Anderson J. 2021 A novel variant in KCNQ1 associated with short QT syndrome. HeartRhythm Case Rep. **7**, 650-654. (10.1016/j.hrcr.2021.04.017) 34712558 PMC8530816

[28] Rothenberg I et al. 2016 Structural interplay of KV7.1 and KCNE1 is essential for normal repolarization and is compromised in short QT syndrome 2 (KV7.1-A287T). HeartRhythm Case Rep. **2**, 521-529. (10.1016/j.hrcr.2016.08.015) 28491751 PMC5420010

[29] Endres D et al. 2020 New Cav1.2 channelopathy with high-functioning autism, affective disorder, severe dental enamel defects, a short QT interval, and a novel CACNA1C loss-of-function mutation. Int. J. Mol. Sci. **21**, 8611. (10.3390/ijms21228611) 33203140 PMC7696251

[30] Zhong R et al. 2022 Epigenetic mechanism of L-type calcium channel beta-subunit downregulation in short QT human induced pluripotent stem cell-derived cardiomyocytes with CACNB2 mutation. Europace **24**, 2028-2036. (10.1093/europace/euac091) 35894107

[31] Walsh R et al. 2022 Evaluation of gene validity for CPVT and short QT syndrome in sudden arrhythmic death. Eur. Heart J. **43**, 1500-1510. (10.1093/eurheartj/ehab687) 34557911 PMC9009401

[32] Suzuki H et al. 2021 Novel electrocardiographic criteria for short QT syndrome in children and adolescents. Europace **23**, 2029-2038. (10.1093/europace/euab097) 34179980

[33] Hu D et al. 2017 The phenotypic spectrum of a mutation hotspot responsible for the short QT syndrome. JACC: Clin. Electrophysiol. **3**, 727-743. (10.1016/j.jacep.2016.11.013) 29759541

[34] Trudeau MC, Warmke JW, Ganetzky B, Robertson GA. 1995 HERG, an inward rectifier in the voltage-gated potassium channel family. Science **269**, 92-95. (10.1126/science.7604285) 7604285

[35] Sanguinetti MC, Jiang C, Curran ME, Keating MT. 1995 A mechanistic link between an inherited and an acquired cardiac arrhythmia: HERG encodes the I_Kr_ potassium channel. Cell **81**, 299. (10.1016/0092-8674(95)90340-2) 7736582

[36] Sanguinetti MC, Tristani-Firouzi M. 2006 hERG potassium channels and cardiac arrhythmia. Nature **440**, 463-469. (10.1038/nature04710) 16554806

[37] Vandenberg JI, Walker BD, Campbell TJ. 2001 HERG K+ channels: friend and foe. TIPS **22**, 240-246. 11339975 10.1016/s0165-6147(00)01662-x

[38] Zhou Z, Gong Q, Ye B, Fan Z, Makielski JC, Robertson GA, January CT. 1998 Properties of HERG channels stably expressed in HEK 293 cells studied at physiological temperature. Biophys. J. **74**, 230-241. (10.1016/S0006-3495(98)77782-3) 9449325 PMC1299377

[39] Hancox JC, Levi AJ, Witchel HJ. 1998 Time course and voltage dependence of expressed HERG current compared with native ‘rapid’ delayed rectifier K current during the cardiac ventricular action potential. Pflugers Archiv – Eur. J. Physiol. **436**, 843-853. (10.1007/s004240050713) 9799397

[40] Ten Tusscher KH, Panfilov AV. 2006 Cell model for efficient simulation of wave propagation in human ventricular tissue under normal and pathological conditions. Phys. Med. Biol. **51**, 6141-6156. (10.1088/0031-9155/51/23/014) 17110776

[41] Wang W, Mackinnon R. 2017 Cryo-EM structure of the open human ether-a-go-go-related K^+^ channel hERG. Cell **169**, 422-430. (10.1016/j.cell.2017.03.048) 28431243 PMC5484391

[42] Sun J, Mackinnon R. 2020 Structural basis of human KCNQ1 modulation and gating. Cell **180**, 340-347. (10.1016/j.cell.2019.12.003) 31883792 PMC7083075

[43] Jumper J et al. 2021 Highly accurate protein structure prediction with AlphaFold. Nature **596**, 583-589. (10.1038/s41586-021-03819-2) 34265844 PMC8371605

[44] Lu Y, Mahautsmith MP, Varghese A, Huang CLH, Kemp PR, Vandenberg JI. 2001 Effects of premature stimulation on HERG channels. J. Physiol. **537**, 843-851. (10.1113/jphysiol.2001.012690) 11744759 PMC2278992

[45] Brugada R et al. 2004 Sudden death associated with short-QT syndrome linked to mutations in HERG. Circulation **109**, 30-35. (10.1161/01.CIR.0000109482.92774.3A) 14676148

[46] Hong K, Bjeerregaard P, Gussak I, Brugada R. 2005 Short QT syndrome and atrial fibrillation caused by mutation in KCNH2. J. Cardivas. Electophysiol. **16**, 394-396. (10.1046/j.1540-8167.2005.40621.x) 15828882

[47] Liu J, Zhang M, Jiang M, Tseng GN. 2002 Structural and functional role of the extracellular S5-P linker in the HERG potassium channel. J. Gen. Physiol. **120**, 723-737. (10.1085/jgp.20028687) 12407082 PMC2229555

[48] Dun W, Jiang M, Tseng GN. 1999 Allosteric effects of mutations in the extracellular S5-P loop on the gating and ion permeation properties of the hERG potassium channel. Pflugers Arch. **439**, 141-149. (10.1007/s004240051138) 10651011

[49] Torres AM et al. 2003 Structure of the HERG K+ channel S5P extracellular linker: role of an amphipathic alpha-helix in C-type inactivation. J. Biol. Chem. **278**, 42 136-42 148. (10.1074/jbc.M212824200) 12902341

[50] Cordeiro JM, Brugada R, Wu YS, Hong K, Dumaine R. 2005 Modulation of I_Kr_ inactivation by mutation N588K in KCNH2: a link to arrhythmogenesis in short QT syndrome. Cardiovas. Res. **67**, 498-509. (10.1016/j.cardiores.2005.02.018) 16039272

[51] Mcpate MJ, Duncan RS, Milnes JT, Witchel HJ, Hancox JC. 2005 The N588K-HERG K+ channel mutation in the ‘short QT syndrome': mechanism of gain-in-function determined at 37(C. Biochem. Biophys. Res. Commun. **334**, 441-449. (10.1016/j.bbrc.2005.06.112) 16011830

[52] Zhang H, Hancox JC. 2004 *In silico* study of action potential and QT interval shortening due to loss of inactivation of the cardiac rapid delayed rectifier potassium current. Biochem. Biophys. Res. Commun. **322**, 693-699. (10.1016/j.bbrc.2004.07.176) 15325285

[53] Priori SG et al. 2005 A novel form of short QT syndrome (SQT3) is caused by a mutation in the KCNJ2 gene. Circ. Res. **96**, 800-807. (10.1161/01.RES.0000162101.76263.8c) 15761194

[54] Itoh H, Horie M, Ito M, Imoto K. 2006 Arrhythmogenesis in the short-QT syndrome associated with combined HERG channel gating defects: a simulation study. Circ. J. **70**, 502-508. (10.1253/circj.70.502) 16565572

[55] Adeniran I, Mcpate MJ, Witchel HJ, Hancox JC, Zhang H. 2011 Increased vulnerability of human ventricle to re-entrant excitation in hERG-linked variant 1 short QT syndrome. PLoS Comput. Biol. **7**, e1002313. (10.1371/journal.pcbi.1002313) 22194679 PMC3240585

[56] Akdis D, Saguner AM, Medeiros-Domingo A, Schaller A, Balmer C, Steffel J, Brunckhorst C, Duru F. 2017 Multiple clinical profiles of families with the short QT syndrome. Europace **20**, f113-f121. (10.1093/europace/eux186) 29016797

[57] Schoenherr R, Heinemann SH. 1996 Molecular determinants for activation and inactivation of HERG, a human inward rectifier potassium channel. J. Physiol. **493**, 635-642. (10.1113/jphysiol.1996.sp021410) 8799887 PMC1159013

[58] Hancox JC, Witchel HJ, Varghese A. 1998 Alteration of HERG current profile during the cardiac ventricular action potential, following a pore mutation. Biochem. Biophys. Res. Commun. **253**, 719-724. (10.1006/bbrc.1998.9837) 9918793

[59] Butler A, Zhang Y, Stuart AG, Dempsey CE, Hancox JC. 2018 Action potential clamp characterization of the S631A hERG mutation associated with short QT syndrome. Physiol. Rep. **6**, e13845. (10.14814/phy2.13845) 30175559 PMC6119704

[60] Sun Y et al. 2011 A novel mutation in the KCNH2 gene associated with short QT syndrome. J. Mol. Cell. Cardiol. **50**, 433-441. (10.1016/j.yjmcc.2010.11.017) 21130771

[61] Giustetto C et al. 2011 Long-term follow-up of patients with short QT syndrome. J. Am. Coll. Cardiol. **58**, 587-595. (10.1016/j.jacc.2011.03.038) 21798421

[62] Lees-Miller JP, Subbotina JO, Guo J, Yarov-Yarovoy V, Noskov SY, Duff HJ. 2009 Interactions of H562 in the S5 helix with T618 and S621 in the pore helix are important determinants of hERG1 potassium channel structure and function. Biophys. J. **96**, 3600-3610. (10.1016/j.bpj.2009.01.028) 19413965 PMC2711401

[63] El Harchi A, Melgari D, Zhang YH, Zhang H, Hancox JC. 2012 Action potential clamp and pharmacology of the variant 1 short QT syndrome T618I hERG K(+) channel. PLoS ONE **7**, e52451. (10.1371/journal.pone.0052451) 23300672 PMC3530446

[64] Du C, Zhang H, Harmer SC, Hancox JC. 2022 Identification through action potential clamp of proarrhythmic consequences of the short QT syndrome T618I hERG ‘hotspot’ mutation. Biochem. Biophys. Res. Commun. **596**, 49-55. (10.1016/j.bbrc.2022.01.057) 35114584 PMC8865743

[65] McPate MJ, Zhang H, Ideniran I, Cordeiro JM, Witchel HJ, Hancox JC. 2009 Comparative effects of the short QT N588K mutation at 37°C on hERG K^+^ channel current during ventricular, Purkinje fibre and atrial action potentials: an action potential clamp study. J. Physiol. Pharmacol. **60**, 23-41. 19439805

[66] Butler A, Zhang Y, Stuart AG, Dempsey CE, Hancox JC. 2019 Functional and pharmacological characterization of an S5 domain hERG mutation associated with short QT syndrome. Heliyon **5**, e01429. (10.1016/j.heliyon.2019.e01429) 31049424 PMC6479114

[67] Redpath CJ, Green MS, Birnie DH, Gollob MH. 2009 Rapid genetic testing facilitating the diagnosis of short QT syndrome. Can. J. Cardiol. **25**, e133-e135. (10.1016/S0828-282X(09)70077-7) 19340359 PMC2706774

[68] Itoh H et al. 2008 A novel KCNH2 interval as a modifier for short QT interval. Int. J. Cardiol. **137**, 83-85. (10.1016/j.ijcard.2008.05.050) 18692916

[69] Barajas Martinez D, Hu D, Gollob M, Antzelevitch C. 2011 Abstract 12845: novel gain-of-function N-terminal KCNH2 mutation associated with the short QT syndrome. *Circulation* **124**, A12845.

[70] Itoh H et al. 2009 Latent genetic backgrounds and molecular pathogenesis in drug-induced long-QT syndrome. Circ. Arrhythm Electrophysiol. **2**, 511-523. (10.1161/CIRCEP.109.862649) 19843919

[71] Wang Q et al. 2014 Gain-of-function KCNH2 mutations in patients with Brugada syndrome. J. Cardiovasc. Electrophysiol. **25**, 522-530. (10.1111/jce.12361) 24400717

[72] Adeniran I, Hancox JC, Zhang H. 2013 *In silico* investigation of the short QT syndrome, using human ventricle models incorporating electromechanical coupling. Front. Physiol. **4**, 166. (10.3389/fphys.2013.00166) 23847545 PMC3701879

[73] Whittaker D, Colman MA, Hancox JC, Zhang H. 2015 *In silico* study of the effects of hERG-linked short QT syndrome on the electrical and mechanical activities of human atrial cells. Proc. Physiol. Soc. **34**, PC163.

[74] Whittaker DG, Ni H, Benson AP, Hancox JC, Zhang H. 2017 Computational analysis of the mode of action of disopyramide and quinidine on hERG-linked short QT syndrome in human ventricles. Front. Physiol. **8**, 759. (10.3389/fphys.2017.00759) 29085299 PMC5649182

[75] McPate MJ, Duncan RS, Hancox JC, Witchel HJ. 2008 Pharmacology of the short QT syndrome N588K-hERG K^+^ channel mutation: differential impact on selected class I and class III antiarrhythmic drugs. Br. J. Pharmacol. **155**, 957-966. (10.1038/bjp.2008.325) 18724381 PMC2597231

[76] Whittaker DG, Hancox JC, Zhang H. 2018 *In silico* assessment of pharmacotherapy for human atrial patho-electrophysiology associated with herg-linked short QT syndrome. Front. Physiol. **9**, 1888. (10.3389/fphys.2018.01888) 30687112 PMC6336736

[77] Heikhmakhtiar AK, Abrha AT, Jeong DU, Lim KM. 2020 Proarrhythmogenic effect of the L532P and N588K KCNH2 mutations in the human heart using a 3D electrophysiological model. J. Korean Med. Sci. **35**, e238. (10.3346/jkms.2020.35.e238) 32715669 PMC7384902

[78] Frea S et al. 2015 New echocardiographic insights in short QT syndrome: more than a channellopathy? Heart Rhythm **12**, 2096-2105. (10.1016/j.hrthm.2015.05.024) 26001507

[79] Hancox JC, Adeniran I, Whittaker DG, Zhang H. 2015 To the editor–altered *in vivo* systolic function in the short QT syndrome anticipated *in silico*. Heart Rhythm **12**, e115. (10.1016/j.hrthm.2015.06.035) 26142296

[80] Frea S, Pidello S, Giustetto C, Scrocco C, Gaitan F. 2015 Author's reply to ‘altered *in vivo* systolic function in the short qt syndrome anticipated *in silico*’. Heart Rhythm **12**, e115-e116. (10.1016/j.hrthm.2015.06.036) 26142294

[81] Tamargo J, Caballero R, Gomez R, Valenzuela C, Delpon E. 2004 Pharmacology of cardiac potassium channels. Cardiovasc. Res. **62**, 9-33. (10.1016/j.cardiores.2003.12.026) 15023549

[82] András V, Tomek J, Nagy N, Virág L, Passini E, Rodriguez B, Baczkó I. 2021 Cardiac transmembrane ion channels and action potentials: cellular physiology and arrhythmogenic behavior. Physiol. Rev. **101**, 1083-1176. (10.1152/physrev.00024.2019) 33118864

[83] Virag L, Iost N, Opincariu M, Szolnoky J, Szecsi J, Bogáts G, Szenohradszky P, Varró A, Papp JG. 2001 The slow component of the delayed rectifier potassium current in undiseased human ventricular myocytes. Cardiovas. Res. **49**, 790-797. (10.1016/S0008-6363(00)00306-0) 11230978

[84] Roden DM. 1998 Taking the ‘idio’ out of ‘idiosyncratic": predicting torsades de pointes. Pacing Clin. Electrophysiol. **21**, 1029-1034. (10.1111/j.1540-8159.1998.tb00148.x) 9604234

[85] Sarkar AX, Sobie EA. 2011 Quantification of repolarization reserve to understand inter-patient variability in the response to pro-arrhythmic drugs: a computational analysis. Heart Rhythm **8**, 1749-1755. (10.1016/j.hrthm.2011.05.023) 21699863 PMC3202650

[86] Banyasz T, Horvath B, Jian Z, Izu LT, Chen-Izu Y. 2011 Sequential dissection of multiple ionic currents in single cardiac myocytes under action potential-clamp. J. Mol. Cell. Cardiol. **50**, 578-581. (10.1016/j.yjmcc.2010.12.020) 21215755 PMC3047417

[87] Banyasz T, Jian Z, Horvath B, Khabbaz S, Izu LT, Chen-Izu Y. 2014 Beta-adrenergic stimulation reverses the I_Kr_-I_Ks_ dominant pattern during cardiac action potential. Pflugers Arch. **466**, 2067-2076. (10.1007/s00424-014-1465-7) 24535581 PMC4138296

[88] Modell SM, Lehmann MH. 2006 The long QT syndrome family of cardiac ion channelopathies: a HuGE review. Genet. Med. **8**, 143-155. (10.1097/01.gim.0000204468.85308.86) 16540748

[89] Bellocq C, van Ginneken AC, Bezzina CR, Alders M, Escande D, Mannens MM, Baró I, Wilde AA. 2004 Mutation in the KCNQ1 gene leading to the short QT-interval syndrome. Circulation **109**, 2394-2397. (10.1161/01.CIR.0000130409.72142.FE) 15159330

[90] El Harchi A, Mcpate MJ, Zhang YH, Zhang H, Hancox JC. 2010 Action potential clamp and mefloquine sensitivity of recombinant ‘I KS’ channels incorporating the V307L KCNQ1 mutation. J. Physiol. Pharmacol. **61**, 123-131. 20436212

[91] Zhang H, Kharche S, Holden AV, Hancox JC. 2008 Repolarisation and vulnerability to re-entry in the human heart with short QT syndrome arising from KCNQ1 mutation–a simulation study. Prog. Biophys. Mol. Biol. **96**, 112-131. (10.1016/j.pbiomolbio.2007.07.020) 17905416

[92] Adeniran I, Whittaker DG, El Harchi A, Hancox JC, Zhang H. 2017 In silico investigation of a KCNQ1 mutation associated with short QT syndrome. Sci. Rep. **7**, 8469. (10.1038/s41598-017-08367-2) 28814790 PMC5559555

[93] Hong K et al. 2005 De novo KCNQ1 mutation responsible for atrial fibrillation and short QT syndrome *in utero*. Cardiovasc. Res. **68**, 433-440. (10.1016/j.cardiores.2005.06.023) 16109388

[94] Villafane J, Fischbach P, Gebauer R. 2014 Short QT syndrome manifesting with neonatal atrial fibrillation and bradycardia. Cardiology **128**, 236-240. (10.1159/000360758) 24818999

[95] Righi D, Silvetti MS, Drago F. 2016 Sinus bradycardia, junctional rhythm, and low-rate atrial fibrillation in short QT syndrome during 20 years of follow-up: three faces of the same genetic problem. Cardiol. Young **26**, 589-592. (10.1017/S1047951115001432) 26279191

[96] Whittaker DG, Colman MA, Ni H, Hancox JC, Zhang H. 2018 Human atrial arrhythmogenesis and sinus bradycardia in KCNQ1-linked short QT syndrome: insights from computational modelling. Front. Physiol. **9**, 1402. (10.3389/fphys.2018.01402) 30337886 PMC6180159

[97] Moreno C et al. 2015 A new KCNQ1 mutation at the S5 segment that impairs its association with KCNE1 is responsible for short QT syndrome. Cardiovasc. Res. **107**, 613-623. (10.1093/cvr/cvv196) 26168993

[98] Nakajo K, Kubo Y. 2014 Steric hindrance between S4 and S5 of the KCNQ1/KCNE1 channel hampers pore opening. Nat. Commun. **5**, 4100. (10.1038/ncomms5100) 24920132

[99] Wu ZJ, Huang Y, Fu YC, Zhao XJ, Zhu C, Zhang Y, Xu B, Zhu QL, Li Y. 2015 Characterization of a Chinese KCNQ1 mutation (R259H) that shortens repolarization and causes short QT syndrome 2. J. Geriatr. Cardiol. **12**, 394-401. 26346102 10.11909/j.issn.1671-5411.2015.04.002 PMC4554793

[100] Rhodes TE et al. 2008 Cardiac potassium channel dysfunction in sudden infant death syndrome. J. Mol. Cell. Cardiol. **44**, 571-581. (10.1016/j.yjmcc.2007.11.015) 18222468 PMC2386856

[101] Arnestad M et al. 2007 Prevalence of long-QT syndrome gene variants in sudden infant death syndrome. Circulation **115**, 361-367. (10.1161/CIRCULATIONAHA.106.658021) 17210839

[102] Mitcheson JS, Hancox JC. 1999 An investigation of the role played by the E-4031-sensitive (rapid delayed rectifier) potassium current in isolated rabbit atrioventricular nodal and ventricular myocytes. Pflugers Archiv - Eur. J. Physiol. **438**, 843-850. (10.1007/s004240051114) 10591073

[103] Gaborit N, Le Bouter S, Szuts V, Varro A, Escande D, Nattel S, Demolombe S. 2007 Regional and tissue specific transcript signatures of ion channel genes in the non-diseased human heart. J. Physiol. **582**(Pt 2), 675-693. (10.1113/jphysiol.2006.126714) 17478540 PMC2075332

[104] Wang Z, Yue L, White M, Pelletier G, Nattel S. 1998 Differential distribution of inward rectifier potassium channel transcripts in human atrium versus ventricle. Circulation **98**, 2422-2428. (10.1161/01.CIR.98.22.2422) 9832487

[105] Perez-Riera AR, Barbosa-Barros R, Samesina N, Pastore CA, Scanavacca M, Daminello-Raimundo R, De Abreu LC, Nikus K, Brugada P. 2021 Andersen-Tawil syndrome: a comprehensive review. Cardiol. Rev. **29**, 165-177. (10.1097/CRD.0000000000000326) 32947483

[106] Abrams CJ, Davies NW, Shelton PA, Stanfield PR. 1996 The role of a single aspartate residue in ionic selectivity and block of a murine inward rectifier K+ channel Kir2.1. J. Physiol. **493**(Pt 3), 643-649. (10.1113/jphysiol.1996.sp021411) 8799888 PMC1159014

[107] Harchi A E, Mcpate MJ, Zhang YH, Zhang H, Hancox JC. 2009 Action potential clamp and chloroquine sensitivity of mutant Kir2.1 channels responsible for variant 3 short QT syndrome. J. Mol. Cell. Cardiol. **137**, 83-85. 10.1016/j.yjmcc.2009.02.027 PMC2765655 19285083

[108] Adeniran I, El Harchi A, Hancox JC, Zhang H. 2012 Proarrhythmia in KCNJ2-linked short QT syndrome: insights from modelling. Cardiovasc. Res. **94**, 66-76. (10.1093/cvr/cvs082) 22308236

[109] Hattori T et al. 2012 A novel gain-of-function KCNJ2 mutation associated with short-QT syndrome impairs inward rectification of Kir2.1 currents. Cardiovasc. Res. **93**, 666-673. (10.1093/cvr/cvr329) 22155372

[110] Yang D et al. 2021 MicroRNA biophysically modulates cardiac action potential by direct binding to ion channel. Circulation **143**, 1597-1613. (10.1161/CIRCULATIONAHA.120.050098) 33590773 PMC8132313

[111] Ambrosini E et al. 2014 Genetically induced dysfunctions of Kir2.1 channels: implications for short QT3 syndrome and autism-epilepsy phenotype. Hum. Mol. Genet. **23**, 4875-4886. (10.1093/hmg/ddu201) 24794859 PMC4140467

[112] Deo M et al. 2013 KCNJ2 mutation in short QT syndrome 3 results in atrial fibrillation and ventricular proarrhythmia. Proc. Natl Acad. Sci. USA **110**, 4291-4296. (10.1073/pnas.1218154110) 23440193 PMC3600465

[113] Meijer Van Putten RME, Mengarelli I, Guan K, Zegers JG, Van Ginneken ACG, Verkerk AO, Wilders R. 2015 Ion channelopathies in human induced pluripotent stem cell derived cardiomyocytes: a dynamic clamp study with virtual I_K1_. Front. Physiol. **6**, 7. (10.3389/fphys.2015.00007) 25691870 PMC4315032

[114] Du C, Rasmusson RL, Bett GC, Franks B, Zhang H, Hancox JC. 2021 Investigation of the effects of the short QT syndrome D172N Kir2.1 Mutation on ventricular action potential profile using dynamic clamp. Front. Pharmacol. **12**, 794620. (10.3389/fphar.2021.794620) 35115940 PMC8806151

[115] Treat JA, Pfeiffer R, Barajas-Martinez H, Goodrow RJ, Bot C, Haedo RJ, Knox R, Cordeiro JM. 2021 Overlap arrhythmia syndromes resulting from multiple genetic variations studied in human induced pluripotent stem cell-derived cardiomyocytes. Int. J. Mol. Sci. **22**, 7108. (10.3390/ijms22137108) 34281161 PMC8268422

[116] Extramiana F, Antzelevitch C. 2004 Amplified transmural dispersion of repolarization as the basis for arrhythmogenesis in a canine ventricular-wedge model of short-QT syndrome. Circulation **110**, 3661-3666. (10.1161/01.CIR.0000143078.48699.0C) 15569843

[117] Frommeyer G, Ellermann C, Dechering DG, Kochhaeuser S, Boegeholz N, Guener F, Leitz P, Pott C, Eckardt L. 2016 Ranolazine and vernakalant prevent ventricular arrhythmias in an experimental whole-heart model of short QT syndrome. J. Cardiovasc. Electrophysiol. **27**, 1214-1219. (10.1111/jce.13029) 27283775

[118] Frommeyer G, Weller J, Ellermann C, Kaese S, Kochhäuser S, Lange PS, Dechering DG, Eckardt L. 2017 Antiarrhythmic properties of ivabradine in an experimental model of short-QT- syndrome. Clin. Exp. Pharmacol. Physiol. **44**, 941-945. (10.1111/1440-1681.12790) 28556923

[119] Patel C, Antzelevitch C. 2008 Cellular basis for arrhythmogenesis in an experimental model of the SQT1 form of the short QT syndrome. Heart Rhythm **5**, 585-590. (10.1016/j.hrthm.2008.01.022) 18362027 PMC2361425

[120] Hassel D et al. 2008 Deficient zebrafish ether-a-go-go-related gene channel gating causes short-QT syndrome in zebrafish reggae mutants. Circulation **117**, 866-875. (10.1161/CIRCULATIONAHA.107.752220) 18250272

[121] Zhang YH, Colenso CK, Sessions RB, Dempsey CE, Hancox JC. 2011 The hERG K^+^ channel S4 domain L532P mutation: characterization at 37 degrees C. Biochim. Biophys. Acta **1808**, 2477-2487. (10.1016/j.bbamem.2011.07.001) 21777565 PMC3245891

[122] Odening KE et al. 2018 Transgenic short-QT syndrome 1 rabbits mimic the human disease phenotype with QT/action potential duration shortening in the atria and ventricles and increased ventricular tachycardia/ventricular fibrillation inducibility. Eur. Heart J. **40**, 842-853. (10.1093/eurheartj/ehy761) 30496390

[123] El-Battrawy I et al. 2018 Modeling short QT syndrome using human-induced pluripotent stem cell-derived cardiomyocytes. J. Am. Heart Assoc. **7**, e007394. (10.1161/JAHA.117.007394) 29574456 PMC5907581

[124] Guo F et al. 2018 Patient specific and gene corrected induced pluripotent stem-cell derived cardiomyocytes elucidate single cell phenotype of short QT syndrome. Circ. Res. **124**, 66-78. (10.1161/CIRCRESAHA.118.313518) 30582453

[125] Shinnawi R et al. 2019 Modeling reentry in the short QT syndrome with human-induced pluripotent stem cell-derived cardiac cell sheets. J. Am. Coll. Cardiol. **73**, 2310-2324. (10.1016/j.jacc.2019.02.055) 31072576

[126] Doss MX et al. 2012 Maximum diastolic potential of human induced pluripotent stem cell-derived cardiomyocytes depends critically on I(Kr). PLoS ONE **7**, e40288. (10.1371/journal.pone.0040288) 22815737 PMC3396384

[127] Hoekstra M, Mummery CL, Wilde AA, Bezzina CR, Verkerk AO. 2012 Induced pluripotent stem cell derived cardiomyocytes as models for cardiac arrhythmias. Front. Physiol. **3**, 346. (10.3389/fphys.2012.00346) 23015789 PMC3449331

[128] Hancox JC, Zhang Y, Du C, Zhang H. 2020 Complementarity between arrhythmia mechanisms found *in silico* and in genetic models of N588K-hERG linked short QT syndrome. J. Integr. Cardiol. Open Access **3**, 1-4. (10.31487/j.JICOA.2020.01.13)

[129] Schimpf R, Wolpert C, Gaita F, Giustetto C, Borggrefe M. 2005 Short QT syndrome. Cardiovasc. Res. **67**, 357-366. (10.1016/j.cardiores.2005.03.026) 15890322

[130] Schimpf R, Wolpert C, Bianchi F, Giustetto C, Gaita F, Bauersfeld U, Borggrefe M. 2003 Congenital short QT syndrome and implantable cardioverter defibrillator treatment: inherent risk for inappropriate shock delivery. J. Cardiovasc. Electrophysiol. **14**, 1273-1277. (10.1046/j.1540-8167.2003.03278.x) 14678099

[131] Schimpf R, Bauersfeld U, Gaita F, Wolpert C. 2005 Short QT syndrome: successful prevention of sudden cardiac death in an adolescent by implantable cardioverter-defibrillator treatment for primary prophylaxis. Heart Rhythm **2**, 416-417. (10.1016/j.hrthm.2004.11.026) 15851347

[132] Dewi IP, Dharmadjati BB. 2020 Short QT syndrome: the current evidences of diagnosis and management. J. Arrhythm **36**, 962-966. (10.1002/joa3.12439) 33335610 PMC7733558

[133] Schwartz PJ, Ackerman MJ, Antzelevitch C, Bezzina CR, Borggrefe M, Cuneo BF, Wilde AAM. 2020 Inherited cardiac arrhythmias. Nat. Rev. Dis. Primers **6**, 58. (10.1038/s41572-020-0188-7) 32678103 PMC7935690

[134] Zeppenfeld K et al. 2022 ESC Guidelines for the management of patients with ventricular arrhythmias and the prevention of sudden cardiac death: The Task Force for the Management of Patients with Ventricular Arrhythmias and the Prevention of Sudden Cardiac Death of the European Society of Cardiology (ESC). Endorsed by: Association for European Paediatric and Congenital Cardiology (AEPC). Eur. Heart J. **43**, 3997-4126. (10.1093/eurheartj/ehac262) 36017572

[135] Gaita F et al. 2004 Short QT syndrome: pharmacological treatment. J. Am. Coll. Cardiol. **43**, 1494-1499. (10.1016/j.jacc.2004.02.034) 15093889

[136] Wolpert C et al. 2005 Further insights into the effect of quinidine in short QT syndrome caused by a mutation in HERG. J. Cardiovasc. Electophysiol. **16**, 54-58. (10.1046/j.1540-8167.2005.04470.x) PMC1474841 15673388

[137] Lees-Miller JP, Duan Y, Teng GQ, Duff HJ. 2000 Molecular determinant of high affinity dofetilide binding to HERG1 expressed in *Xenopus* oocytes: involvement of S6 sites. Mol. Pharmacol. **57**, 367-374. 10648647

[138] Perrin MJ, Kuchel PW, Campbell TJ, Vandenberg JI. 2008 Drug binding to the inactivated state is necessary but not sufficient for high-affinity binding to human ether-à-go-go-related gene channels. Mol. Pharmacol. **74**, 1443-1452. (10.1124/mol.108.049056) 18701618

[139] McPate MJ, Duncan RS, Witchel HJ, Hancox JC. 2006 Disopyramide is an effective inhibitor of mutant HERG K^+^ channels involved in variant 1 short QT syndrome. J. Mol. Cell. Cardiol. **41**, 563-566. (10.1016/j.yjmcc.2006.05.021) 16842817

[140] Luo C, Whittaker DG, Liu T, Wang K, Li Y, He Y, Zhang H. 2019 Pharmacotherapeutic effects of quinidine on short QT syndrome by using Purkinje-Ventricle model: a simulation study. Annu. Int. Conf. IEEE Eng. Med. Biol. Soc. **2019**, 2856-2859. 31946488 10.1109/EMBC.2019.8857134

[141] Villafane J et al. 2013 Long-term follow-up of a pediatric cohort with short QT syndrome. J. Am. Coll. Cardiol. **61**, 1183-1191. (10.1016/j.jacc.2012.12.025) 23375927

[142] Hancox JC, Stuart AG, Harmer SC. 2020 Functional evaluation of gene mutations in long QT syndrome: strength of evidence from *in vitro* assays for deciphering variants of uncertain significance. J. Congenit. Cardiol. **4**, 1-13. (10.1186/s40949-020-00037-9)

[143] Bett GCL, Kaplan AD, Lis A, Cimato TR, Tzanakakis ES, Zhou Q, Morales MJ, Rasmusson RL. 2013 Electronic ‘expression’ of the inward rectifier in cardiocytes derived from human-induced pluripotent stem cells. Heart Rhythm **10**, 1903-1910. (10.1016/j.hrthm.2013.09.061) 24055949 PMC3851822

[144] El-Battrawy I et al. 2018 Long-term follow-up of patients with short QT syndrome: clinical profile and outcome. J. Am. Heart Assoc. **7**, e010073. (10.1161/JAHA.118.010073) 30571592 PMC6405569

[145] Esfandyari D et al. 2022 MicroRNA-365 regulates human cardiac action potential duration. Nat. Commun. **13**, 220. (10.1038/s41467-021-27856-7) 35017523 PMC8752767

[146] Gruber A et al. 2021 Optogenetic modulation of cardiac action potential properties may prevent arrhythmogenesis in short and long QT syndromes. JCI Insight **6**, e147470. (10.1172/jci.insight.147470) 34100384 PMC8262308

[147] El-Battrawy I et al. 2020 Sex-differences in short QT syndrome: a systematic literature review and pooled analysis. Eur. J. Prev. Cardiol. **27**, 1335-1338. (10.1177/2047487319850953) 31122038 PMC7391477

